# Unusually High Archaeal Diversity in a Crystallizer Pond, Pomorie Salterns, Bulgaria, Revealed by Phylogenetic Analysis

**DOI:** 10.1155/2016/7459679

**Published:** 2016-11-16

**Authors:** Margarita Kambourova, Iva Tomova, Ivanka Boyadzhieva, Nadja Radchenkova, Evgenia Vasileva-Tonkova

**Affiliations:** Institute of Microbiology, Bulgarian Academy of Sciences, Acad. G. Bonchev Str. 26, 1113 Sofia, Bulgaria

## Abstract

Recent studies on archaeal diversity in few salterns have revealed heterogeneity between sites and unique structures of separate places that hinder drawing of generalized conclusions. Investigations on the archaeal community composition in P18, the biggest crystallizer pond in Pomorie salterns (PS) (34% salinity), demonstrated unusually high number of presented taxa in hypersaline environment. Archaeal clones were grouped in 26 different operational taxonomic units (OTUs) assigned to 15 different genera from two orders, Halobacteriales and Haloferacales. All retrieved sequences were related to culturable halophiles or unculturable clones from saline (mostly hypersaline) niches. New sequences represented 53.9% of archaeal OTUs. Some of them formed separate branches with 90% similarity to the closest neighbor. Present results significantly differed from the previous investigations in regard to the number of presented genera, the domination of some genera not reported before in such extreme niche, and the identification of previously undiscovered 16S rRNA sequences.

## 1. Introduction

Solar salterns are designed for production of common salt (NaCl) from coastal sea water and they differ in their salt concentration, chemical composition, and geographic location. They represent extreme habitats that favor growth of extreme halophiles (optimal growth above 15% NaCl), while moderate halophiles (optimal growth 3–15% NaCl) and slight halophiles (1–3% NaCl) are not able to grow at such environments [1]. Archaeal representatives dominate in solar salterns. The high salt concentration is the main factor affecting diversity in hypersaline environments because the number of microbial species decreases with the increasing salinity, and a few taxa become dominant [2].

It is commonly accepted that culture dependent methods describe only a small part of real diversity in natural environments [3] and 16S rRNA analysis of environmental DNA sample has proved to be a powerful approach of microbial identification and evaluation of diversity. In the last two decades several studies have been performed on diversity in coastal solar salterns in different geographic areas including Tunisia, Israel, Australia, Mexico, and India [2, 4–8]. In Europe, hypersaline microbiota has been intensively investigated in coastal salterns located in Spain [9–11] and Croatia [12]. These studies have revealed community heterogeneity between sites that have repeatedly been reported over the years [2, 6, 12, 13]. The observed differences could be explained by the restricted dispersal at long geographic distance, and in such a way evolutionary events could give rise to diversity in populations from separate geographic locations and unique lineages could appear [14]. An existence of many novel taxa in the salterns has been suggested by several authors [1, 6, 10, 15]. Additionally, nutrient levels or other unidentified environmental factors might be responsible for microbiota variety [16].

Comparison of the results reported by some authors has revealed that archaeal communities in solar salterns are rather similar at the phylum level, but there are only few cosmopolitan taxa at lower taxonomic level. The square archaeon* Haloquadratum waslbyi* and a new* Candidate* archaeal class, Nanohaloarchaea, have been reported as most common in the archaeal communities [16, 17]. Metagenomic studies on biodiversity in ponds with different salinity in Santa Pola saltern have shown that the only phylum shared by a crystallizer pond (37% NaCl) [18] and intermediate-salinity pond (13%) [19] is Euryarchaeota and it dominates at higher salinity.

To the best of our knowledge archaeal community structure in coastal salterns from the area of Black Sea coast has not been characterized. The aim of the current work was to use 16S rRNA gene analysis to investigate archaeal diversity in the biggest crystallizer pond in Pomorie salterns (PS), P18, and to compare it with community structure in crystallizers from coastal solar salterns worldwide.

## 2. Materials and Methods

### 2.1. Sampling Site

The coastal lagoon Pomorie salterns (42.63N, 27.62E) is located north of the town of Pomorie, West Black Sea cost. The lake is separated from the sea with natural sand and artificial dike and a connecting channel is available only in the southern part, which is implemented by the inflow and outflow of seawater. Its area is about 8–8,5 km^2^, length is of 5-6 km, the width varies from 350 m north to 1,6 km in the middle part, and depth is not greater than 1,4 m. Temperatures are moderate, with average July temperature of 24°C and January temperature of 2.7°C, and annual rainfall is 598 mm/year. They are typical multipond salterns with a discontinuous salinity gradient up to saturation used for the extraction of salt (about 30,000 tons per year) and healing mud. Sampling site was the biggest crystallizer pond P18, 350 × 400 m with a salinity of 340 g L^−1^, belonging to the so-called thalassohaline environments.

Brine water was collected aseptically in June 2014 from ten different sites of the crystallizer pond PS18 in order to obtain a representative sample. The homogenized sample was transported in a cooler bag to the lab and stored at −4°C prior to the initiation of the procedure for DNA isolation. The chemical and physical properties of the water sample were analyzed by a commercial water chemistry laboratory DIAL Ltd., Bulgaria. The analysis of water from the crystallizer pond P18 showed the following ion composition (g L^−1^): Cl^−^, 188.38; SO_4_^2−^, 26.59; Na^+^, 101.10; Mg^2+^, 18.02; K^+^, 6.21; Ca^2+^, 0.32; B^3+^, 0.076; Sr^2+^, 0.022. The total salt concentration was 34%, pH 7.8, EC (mS cm^−1^) 197.6.

### 2.2. DNA Isolation, PCR Amplification, and Construction of 16S rRNA Libraries

Total DNA was extracted from the sediment RB sample as described by Selenska-Pobell et al. [20] with some modifications. A sample (3 L) was concentrated by cross-flow filtration through sterile hollow fiber cartridges (1.2 *μ*m pore-size glass fiber prefilter and 0.2 *μ*m membrane filter; Millipore). The filter was stored at −20°C for subsequent DNA extraction. The sample material was suspended in 10 mL of 0.12 M sodium phosphate buffer. Lysis of the cells was achieved after adding sodium dodecyl sulfate (final concentration 2%), NaCl (0.5 M), and PEG 6000 (20%). The protocol for extraction of a total community DNA encompassed three cycles of freezing and thawing (correspondingly −80°C and 96°C), chemical lysis in an extraction buffer, and a proteinase K step. The crude DNA was purified with the AXG-100 Nucleobond cartridges (Machery-Nagel, Duren, Germany) following the manufacturer's instructions. The eluate was precipitated by 0.7 volumes of ice-cold isopropanol. A total amount of 45 *μ*g DNA was extracted from the sample. The integrity of the DNA was checked by horizontal electrophoresis in 1% agarose (Sigma) gel and visualized with ethidium bromide (0.5 mg L^−1^).

The extracted genomic DNA was used as a target for PCR amplification of 16S rRNA genes. Community ribosomal DNAs were amplified from 1 to 50 ng of bulk DNA in reaction containing (as final concentrations) 1x PCR buffer, 2 mM CaCl_2_, 4x 200 *μ*M deoxynucleoside triphosphates, 400 nM each forward and reverse primer, and 0.5 U Taq polymerase (GenetBio, Korea). The archaeal 16S rDNA specific primers 21F (5′-TTCCGGTTGATCCYGCCGGA-3′) and 958R (5′-YCCGGCGTTGCCAATT-3′) [21] were used for amplification. Reaction mixtures were incubated in BioRad thermal cycler T100 using an initial denaturation at 94°C for 3 min, followed by 30 cycles of 94°C for 30 sec, 55°C for 30 sec, and 72°C for 1 min and a final extension at 72°C for 20 min.

The PCR products were cloned in* E. coli* JM 109 using pJet1.2 cloning kit (Fermentas) according to the manufacturer's instructions. Cloned fragments were reamplified using pJet1.2 forward and reverse primers located in the vector and surrounding the inserted PCR fragment.

### 2.3. Analysis of the Library and Clone Selection

Screening of the library was conducted with two separate RFLP (restriction fragment length polymorphism) analyses. For obtaining the highest resolution of RFLP analysis, four base restriction enzymes were used. Ten *μ*L of the reamplified PCR products was separately digested with 5 U of each endonuclease, Msp I, and Hae III (Fermentas) in a final volume of 20 *μ*L for 2 h at 37°C according to the manufacturer's instructions (Fermentas). The generated fragments were separated on a 2% agarose gel. Restriction fragments shorter than 100 bp were not considered in the analysis. Bands were visualized by staining with ethidium bromide and UV illumination. The clones with the same restriction patterns (band pattern characteristic of the restricted PCR product) were grouped in one OTU. At least one clone per a restriction pattern was sequenced.

### 2.4. 16S rRNA Gene Sequencing and Analysis

16S rRNA gene sequences were determined with Applied Biosystems model 373A DNA sequencer by using the ABI PRISM cycle sequencing kit (Macrogen, Netherlands), where they were reamplified by using the above primers. 16S rRNA gene sequences were initially compared with reference sequences at NCBI (https://www.ncbi.nlm.nih.gov/) using BLAST [22] and Ribosomal Database Project resources [23] to determine their close relatives and approximate phylogenetic affiliations. Phylogenetic analysis was conducted using MEGA version 6.0 [24] and neighbor-joining method [25]. Cloned sequences were checked for possible chimeric structures using the program Chimera Check at the Ribosome Database Project website (http://rdp.cme.msu.edu/) and the established five chimera sequences were excluded from further analysis. Those of the clones that showed less than 97% similarity to the closest relative after sequencing were referred to as new.

The degree of the diversity in the sample was measured with both, cover analysis [26] in which coverage value was derived from the equation *C* = 1 − (*n*/*N*) × 100, where *n* was the number of unique clones and *N* was the total number of the examined clones, and the Shannon index [27]: *H* = −sum_*i*_(*p*_*i*_ln⁡(*p*_*i*_)), where *p*_*i*_ was the relative frequency of the *i*th clone among the examined clones and ln was the natural logarithm.

### 2.5. Nucleotide Sequence Accession Numbers

The 16S rRNA gene sequences reported in this study were submitted to EMBL, GenBank databases under accession numbers LN865027 to LN865053.

## 3. Results and Discussion

**Figure 1:**
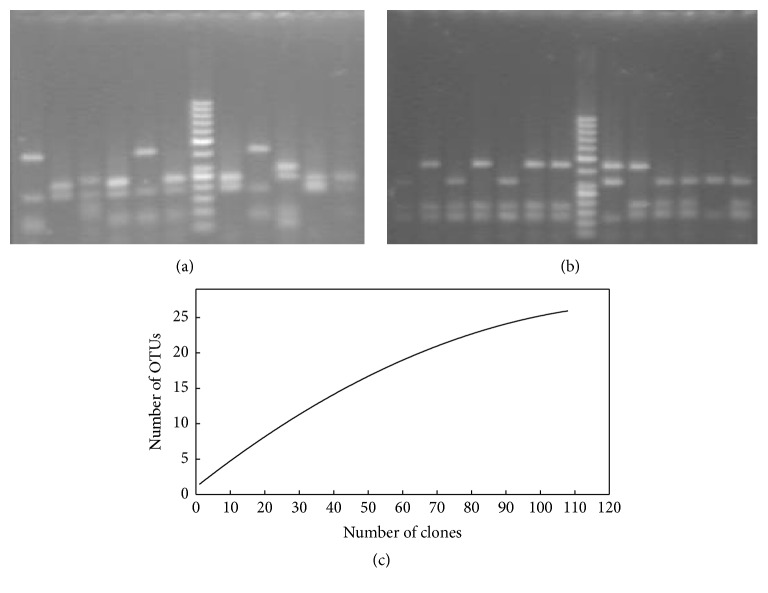
RFLP analysis of the retrieved clones. (a) Agarose gel electrophoresis of products after restriction with Msp I; 1–6, 8–12: restriction patterns; 7: marker Gene Ruler 50 bp (Fermentas). (b) Agarose gel electrophoresis after restriction with Hae III of the sequences with the same Msp I restriction pattern; 1–6, 8–14: restriction patterns; 7: marker Gene Ruler 50 bp (Fermentas). Additional splitting of the group was observed. (c) Rarefaction curve for sampling of PS 16S rRNA archaeal gene library. Rarefaction curve was calculated using Analytical Rarefaction version 1.3 (http://www.uga.edu/strata/software/index.html).

Totally 112 archaeal clones contained inserts of the expected size of approx. 950 bp. Three of them showed chimeric structures and the rest 109 were selected for further RFLP analysis with Msp I (Figure 1(a)) and Hae III (Figure 1(b)). Restriction products were observed as 3 to 10 bands from digestion of each rDNA and discernible fragment size ranged from 100 to 700 bp. The clones with identical patterns for both restriction enzymes were grouped in 26 discrete OTUs (Table 1). Diversity coverage value *C* was 86% and Shannon index *N* was 5.24. Ten of the sequences were singletons presented only once in the clone library. In order to evaluate whether the number of the restricted clones was sufficient to evaluate diversity within the clone library, rarefaction analysis was applied. Rarefaction curve obtained by plotting the number of OTUs was observed against the number of clones sequenced (Figure 1(c)). A decrease in the detection rate of OTUs was observed with increasing the number of the restricted clones. Although a clear plateau was not observed that demonstrated the richness of the clone libraries and a possibility for revealing of further diversity after additional analysis, this decrease indicated that the major fraction of the diversity in this library was detected.

**Figure 2:**
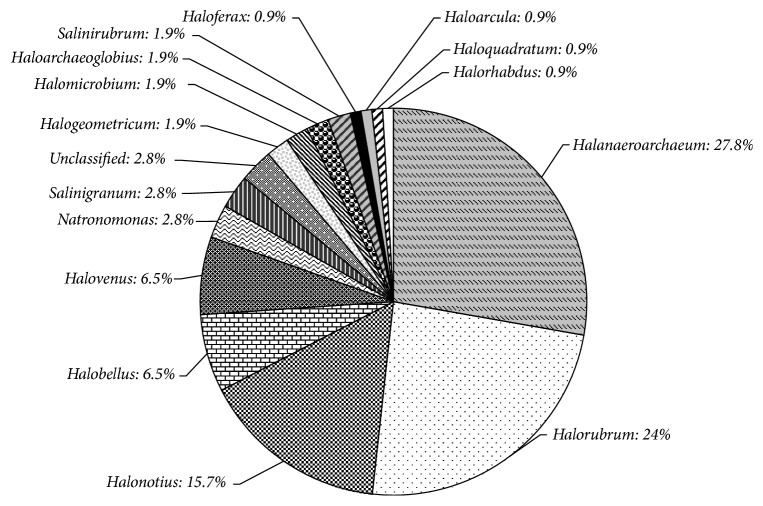
Genus affiliation of 16S rDNAs clones obtained from Pomorie salterns. Sequences were classified using BLAST search results and phylogenetic analysis in ARB.

Fifteen of the described 50 halophilic archaeal genera [28] were found in Pomorie salterns (Figure 2). The detection of unexpected high number genera differed from the common opinion for low diversity of microbiota in hypersaline environments close to saturation and domination of only one cluster [6, 10]. It was significantly higher than that observed by other authors for hypersaline salterns with higher than 30% salt content of the sampling site (Table 2): two in Maras salterns, Peru [13]; four in 32% salt pond, Santa Pola salterns, Spain [10]; four in 30% salt ponds, Guerrero Negro saltern, Mexico [7]; three in 31% salt pond S5 from a solar saltern in Tunisia [2]; nine in Bengal Bay salterns, India [8], revealing that the number of the identified genera in different salterns varies in a wide range from two to nine and fifteen and even higher diversity could be expected having in mind the number of halophilic genera. Comparison of the results for affiliation of the retrieved sequences revealed that archaeal communities are rather similar at higher taxonomic level like family but they differ at lower taxonomic level like genus what could be explained by the above-mentioned endemism in separate geographic locations [14]. Representatives of the same one or two families could be found in all investigated salterns (Table 2) but the dominant genera differed.* Halorubrum* was found to dominate in four of the compared six salterns (including Pomorie),* Haloquadratum* in half of them, and at the same time the genera* Natrinema*,* Halogeometricum,* and* Haloferax* were identified as dominant only in Bengal Bay, India [8],* Halobacterium* only in Maras salterns [13], and* Halanaeroarchaeum* and* Halonotius* only in Pomorie salterns. This is the first report for a domination of* Halanaeroarchaeum* (four OTUs, 28% of all clones) in hypersaline environment followed in frequency by sequences related to four* Halorubrum* OTUs (four different OTUs, 23% of all clones) and* Halonotius* (one OTU, 16%). A domination of* Halorubrum* has been reported for various salterns and salt lakes where* Haloquadratum* is neither absent nor dominant [6, 12, 29] but both genera were determined as dominant in S5, Tunisian solar saltern [2]. Clones affiliated with the cosmopolitan square archaeon* Haloquadratum* have been most frequently retrieved in the clone libraries from crystallizers, coastal salterns [2, 6, 7, 9, 10, 13, 30]. It was represented by only one clone in the library from PS. Other twelve species represented in PS were rarely or not identified in hypersaline environments.

**Figure 3:**
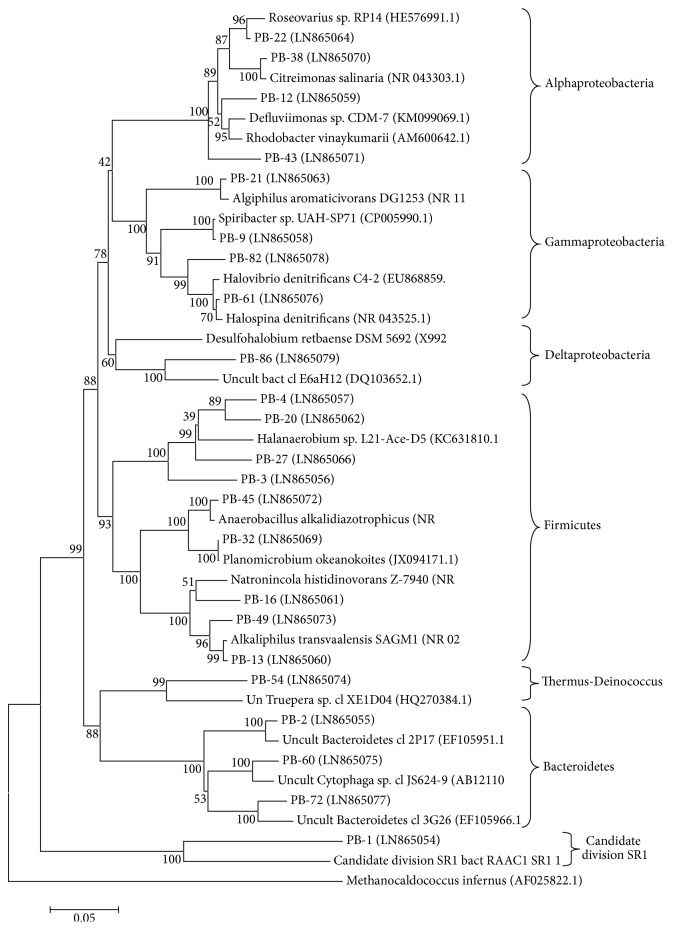
Neighbor-joining phylogenetic tree based on archaeal 16S rDNA sequences found in a crystallizer pond, PS. Bar, 5% substitutions in nucleotide sequence. Bootstrap values greater than 70% confidences are shown at branching points (percentage of 1000 resamplings). Sequence accession numbers are given in parenthesis. A sequence from the thermoacidophilic archaeon* Sulfolobus acidocaldarius* that belongs to the phylum Crenarchaeota constituted the out group.

Similar to other investigations on diversity in saline niches [5, 10, 31] all recovered archaeal sequences from PS were referred to the phylum Euryarchaeota in the kingdom Euryarchaeota and none of them was related with Crenarchaeota or other Proteoarchaeota phyla (according to the classification suggested by Petitjean et al. [32]). The retrieved sequences referred to two of the three validly published haloarchaeal orders in the phylum Euryarchaeota [33], namely, Halobacteriales and Haloferacales with predomination of Halobacteriales (22 OTUs) (Figure 3).

As a common only representatives of the family Halobacteriaceae have been identified by culture independent investigations. Recovering of sequences related with* Haloferax* has been also reported for salterns in Bengal Bay, India [8]. Placing of Haloferaceae OTUs among Halobacteriaceae OTUs in the phylogenetic tree reflects the complicated phylogeny of halophilic arcahaea and confirms the obvious need for further revision of the vast polyphyletic order Halobacteriales [28]. Based on exploration of several different markers the above authors have suggested a division of the order Haloferacales into two families, families Haloferacaceae and Halorubraceae, and a division of the order Halobacteriales into three families, Halobacteriaceae, Haloarculaceae, and Halococcaceae.

All retrieved archaeal sequences grouped in the phylogenetic tree with culturable halophiles (9 OTUs) or unculturable clones from saline (mostly hypersaline) niches worldwide (17 OTUs) such as coastal salterns and hypersaline lakes. Unculturable matches were clones recovered from saline niches worldwide: Salton Sea, US; Lake Kasin, Southern Russia; Cabo de Gata, Spain; Sfax salterns, Tunisia; Salt Spring, Canada; solar salterns, Adriatic Sea; Zhoushan saltern, China; salt lake, Xinjiang, China. Among them four archaeal OTUs were most similar to the sequences from crystallizer ponds 11 and 12, Lake Kasin, Russia [34] but a strong similarity in microbial community composition was not found. A total of six archaeal OTUs were affiliated with ≥97% sequence similarity with cultivated species. All closest culturable relatives were halophilic microorganisms or halotolerants unlike other hypersaline environments in which halophiles and nonhalophilic relatives were found together [13, 31].

Although the investigated environment was among salterns with a highest deal of new sequences as more than a half of the OTUs from the archaeal library (53.9%) showed similarity with the closest match less than 97% the phylogenetic distance of the sequences was no more than 10% suggesting a presence of lower level new taxa. Among the most distantly related clones was PA-80 (Pomorie-Archaea library, clone 80) with 90% similarity to* Haloferax denitrificans*, and PA-26 and PA-112 were grouped independently of any known cultivated haloarchaeon with a modest similarity among them (92%).

## 4. Conclusions

The present investigation revealed that a hypersaline pond from Pomorie salterns harbored novel archaeal diversity that has never been reported before for crystallizers. Community structure differed from other crystallizer ponds in coastal solar salterns in its high number of genera and a presence of halophilic genera that so far had not been considered characteristic for other salterns. Many of them were grouped with unculturable representatives of the corresponding taxa. More than a half of the retrieved sequences were referred to as new, some of them showing phylogenetic distance of more than 10%, suggesting a presence of novel taxonomic divisions higher than species level. The obtained results have important implications for extending the view on microbial diversity in hypersaline environments in new directions and contribute to the development of knowledge for microbiota in salterns.

## Figures and Tables

**Table 1:** Frequencies of OTUs within the Archaea domain derived from the 16S rRNA sequences from а crystallizer pond from Pomorie saltern, Bulgaria. Novel sequences (more than 3% phylogenetic distance), their relative abundance, their closest phylogenetic neighbors, and similarity among them are underlined.

OTU	Accession number (seq. length, bp)	Number of clones	Cluster affiliation	Results of BLAST analysis: closest match, (sequence number), [identity], (isolation source)
**PA-30**	**LN865035**	**20**	Halobacteriaceae, *Halanaeroarchaeum*	Uncultured haloarchaeon clone DE09024B01, KF591560.1, **94%**, Extreme Hypersaline Meromictic Lake
PA-73	LN865045	5	Halobacteriaceae, *Halanaeroarchaeum*	Uncultured euryarchaeote clone DSFBPENV12arc_1E, KC465594.1, 98%, hypersaline pools, Salton Sea
**PA-105**	**LN865050**	**4**	Halobacteriaceae, *Halanaeroarchaeum*	Uncultured archaeon clone Kasin-A1-C11, HE604442.1, **93%**, Hypersaline Lake Kasin, Southern Russia
**PA-21**	**LN865033**	**1**	Halobacteriaceae, *Halanaeroarchaeum*	*Halanaeroarchaeum sulfurireducens* strain HSR2, CP008874.1, **96%**
PA-2	LN865027	17	Halobacteriaceae, *Halorubrum*	*Halorubrum ezzemoulense* CECT 7099, NR_113217.1, 98%
PA-103	LN865049	4	Halobacteriaceae, *Halorubrum*	*Halorubrum* sp. s5a-3, JN196466.1, 98%, saltern Cabo de Gata, Spain
PA-53	LN865040	3	Halobacteriaceae, *Halorubrum*	*Halorubrum californiense* LV_12B39, LN649806.1, 99%
**PA-52**	**LN865039**	**1**	Halobacteriaceae, *Halorubrum*	*Halorubrum orientale* strain EJ-52, NR_042510.1, **94%**
PA-3	LN865028	17	Halobacteriaceae, *Halonotius*	Uncultured archaeon clone TSHNAA23, HQ157628.1, 98%, Sfax salterns, Tunisia
PA-61	LN865043	1	Halobacteriaceae, *Halonotius*	Uncultured archaeon clone SFE1F061, CU467145.1, 98%, solar saltern, Tunisia
PA-8	LN865029	7	Halobacteriaceae, *Halovenus*	Uncultured archaeon 16S rRNA gene, clone ss_048, AJ969840.1 97%, Salt Spring, British Columbia, Canada
**PA-51**	**LN865038**	**3**	Halobacteriaceae, *Natronomonas*	Uncultured *Natronomonas* sp. clone 209ZA09, FN391194.1, **93%**, Sfax salterns, Tunisia
**PA-139**	**LN865052**	**1**	Halobacteriaceae, *Natronomonas*	Uncultured haloarchaeon clone Ston16S367, DQ889328.1, **93%**, solar salterns, Adriatic Sea
PA-11	LN865031	2	Halobacteriaceae, *Salinigranum*	Halobacteriaceae archaeon ZS-5, KJ689293.1, 99%, Zhoushan marine solar salter, China
PA-54	LN865041	1	Halobacteriaceae, *Salinigranum*	Uncultured archaeon clone 1, HE604940.1, 98%, Hypersaline Lake Kasin, Southern Russia
**PA-79**	**LN865046**	**2**	Halobacteriaceae, *Halomicrobium*	Uncultured archaeon clone Kasin-A1-A06, HE604415.1, **93%**, Hypersaline Lake Kasin, Southern Russia
**PA-63**	**LN865044**	**2**	Halobacteriaceae, *Halogeometricum*	*Halogeometricum rufum* isolate LV_13S50, LN649947.1, **93%**
PA-57	LN865042	2	Halobacteriaceae, *Haloarchaeobius*	Uncultured haloarchaeon clone XKL11, JN714414.1, 98%, salt lake, Xinjiang, China
PA-33	LN865036	2	Halobacteriaceae, *Salinirubrum*	*Salinirubrum litoreum* strain YJ-63-S1, KC918824.2, 99%
PA-87	LN865048	1	Halobacteriaceae, *Haloarcula*	*Haloarcula japonica* strain JCM7785, NR_116082.1, 99%
**PA-17**	**LN865032**	**1**	Halobacteriaceae, *Haloquadratum*	Uncultured archaeon clone Kasin-A3-D05, HE604599.1, **96%**, Hypersaline Lake Kasin, Southern Russia
**PA-145**	**LN865053**	**1**	Halobacteriaceae, *Halorhabdus*	Uncultured *Halorhabdus* sp. clone SFH1C101, FN391257.1, **94%**, solar saltern, Tunisia

**PA-89**	**LN865030**	**7**	Haloferaceae, *Halobellus*	*Halobellus litoreus* strain JCM 17118, NR_125476.1, **96%**
**PA-80**	**LN865047**	**1**	Haloferaceae, *Haloferax*	*Haloferax denitrificans* strain JCM 8864, NR_113439.1, **90%**

**PA-26**	**LN865034**	**2**	Unclassified	Uncultured euryarchaeote clone DSFBPENV12arc_7G, KC465577.1, **95%**, hypersaline pools in the Salton Sea, US
**PA-112**	**LN865051**	**1**	Unclassified	Uncultured archaeon clone ss_014, AJ969886.1, **95%**, Salt Spring, British Columbia, Canada

**Table 2:** Comparison of the archaeal diversity in Pomorie saltern and other thalassohaline hypersaline ecosystems.

Saltern	Additional environmental factors	Number of OTUs presented	Division presented, %	Number of genera presented	Dominant genera^a^, %	New sequences^b^, %	Reference
Pomorie	Temp. 24°C, annual rainfall 598 mm/year, pH 7.2, organic carbon 190 mg/L	27	Halobacteriaceae Haloferacaceae	15	*Halanaeroarchaeum*, 28, *Halorubrum*, 23, *Halonotius*, 17	53.9	This study
Maras salterns	pH 6.5–7.0	6	Halobacteriaceae	2	*Haloquadratum*, 69, *Halobacterium*, 31	33.3	[13]
Guerrero Negro saltern (ponds with more than 30% salt)	Temp. 16.2–18.9°C	19	Halobacteriaceae	4	*Haloquadratum*, >60%	n.d.	[7]
Tunisian solar saltern, S5	Annual rainfall 230 mm/year average temperature of 15 and 33°C for the hottest and coldest months, respectively, pH 7.4	40	Halobacteriaceae	3	*Haloquadratum*, 55, *Halorubrum*, 36	66.7	[2]
Santa Pola salterns (32% salt pond)	Temp. 28°C	11	Halobacteriaceae	4	*Halorubrum*	n.d.	[10]
Three salterns, Bengal Bay, India	pH 7.22–7.89 Temperature 32.1–36.6	10	Halobacteriaceae Haloferacaceae	9	*Natrinema*, 32.4%, *Halorubrum*, 19.1%, *Halogeometricum*, 11.8%, *Haloferax*, 11.8%	0	[8]

^a^Phylogenetic groups representing more than 10% of the community sequences were determined as dominant.

^b^Sequences with less than 97% similarity to the closest neighbor were referred to as new sequences.
